# miR-101-3p improves neuronal morphology and attenuates neuronal apoptosis in ischemic stroke in young mice by downregulating HDAC9

**DOI:** 10.1515/tnsci-2022-0286

**Published:** 2023-05-26

**Authors:** Mengru Zhang, Jianjun Wang, Jinfang Li, Fanxin Kong, Songjun Lin

**Affiliations:** The Fourth Clinical Medical College of Guangzhou University of Chinese Medicine, Shenzhen, 518000, China; Encephalopathy and Psychology Department, Shenzhen Traditional Chinese Medicine Hospital, Shenzhen, 518000, China

**Keywords:** ischemic stroke, miR-101-3p, HDAC9, apoptosis, neurons, children

## Abstract

**Objective:**

MiRNAs play a key role in ischemic stroke (IS). Although miR-101-3p can participate in multiple disease processes, its role and mechanism in IS are not clear. The aim of the present study was to observe the effect of miR-101-3p activation on IS in young mice and the role of HDAC9 in this effect.

**Methods:**

The young mice were first subjected to transient middle cerebral artery occlusion (tMCAO) or sham surgery, and the cerebral infarct area was assessed with 2,3,5-triphenyltetrazolium chloride staining. Meanwhile, the expressions of miR-101-3p and HDAC9 were tested using RT-qPCR or western blot. Besides, neuron morphology and apoptosis were confirmed using Nissl staining and TUNEL staining.

**Results:**

We first verified that miR-101-3p was downregulated and HDAC9 was upregulated in the brain tissue of tMCAO young mice. Moreover, we proved that overexpression of miR-101-3p could improve cerebral infarction, neuronal morphology, and neuronal apoptosis in tMCAO young mice by lowering the expression of HDAC9.

**Conclusions:**

Activation of miR-101-3p can protect against IS in young mice, and its mechanism is relevant to the inhibition of HDAC9. Therefore, miR-101-3p and HDAC9 might be the latent targets for IS therapy.

## 1 Introduction

Stroke is the main cause of death and disability in adults, but its incidence is not limited to adults [1]. The incidence of stroke in children is low and the clinical manifestations are diverse, but the mortality and disability rates are high [2]. The annual incidence of stroke in children is about 1/100,000 to 6/100,000, and the incidence of stroke in perinatal children is higher, about 1/3,500 [3]. With the development of medical imaging, the detection rate of stroke in children has increased and its incidence is similar to that of tumor diseases [4]. The international incidence of ischemic stroke (IS) in children is (0.06–0.79)/10,000. The clinical features, risk factors, pathophysiology, response to reperfusion, and treatment strategies of IS in children are different from those in adults [5,6]. Early diagnosis and remedy are crucial for both adults and children. In children, there is a significant delay in the diagnosis of IS, with an average delay of 22.7–24.8 h, which is longer than the optimal treatment time (6 h) [7].

MicroRNAs (miRNAs) are short non-coding RNAs commonly discovered in the post-transcriptional regulation of gene expression [8,9]; miRNA is highly conserved evolutionarily and has tissue specificity and timeliness [10]. Many studies have verified that miRNAs are related to the occurrence and development of tumors, cardiovascular diseases, diabetes, neurologic disorder, and genetic diseases [11,12,13,14]. In addition, miRNA plays a key role in ontogeny, energy metabolism, cell proliferation, apoptosis, etc. MiRNA expression often changes abnormally in IS patients, and certain miRNAs, such as miR-126 [15], miR-191 [16], miR-532-5p [17], and miR-130a [18], have been confirmed as markers for IS. The existing studies proved that miR-101-3p was relevant to the apoptosis of oral cancer [19], irradiation sensitivity of lung cancer [20], oxaliplatin sensitivity of hepatocellular carcinoma [21], and proliferation and metastasis of breast cancer [22]. Besides, miR‑101‑3p was lowly expressed in glioblastoma cells, and it also could block the proliferation and metastasis of glioblastoma cells [23]. Above all, new studies showed that miR-101-3p could evidently alleviate myocardial injury induced by sepsis [24], and it was connected with neuroinflammation and neuronal injury in the spinal cord [25]. miR-101-3p also had an obvious protective role in testicular ischemia reperfusion injury (IRI) through induction of antioxidation [26]. However, the impact and mechanism of miR-101-3p in children with IS are not completely clear. Among the 15 miRNAs considered, miR-101a-3p emerges as the most promising IS biomarker for further future evaluation [27].

In this study, we established transient middle cerebral artery occlusion (tMCAO) model in young mice and further observed the expression of miR-101-3p in tMCAO young mice. Functionally, we further verified the influence of miR-101-3p overexpression on cerebral infarction, neuronal morphology, and apoptosis in tMCAO young mice. Furthermore, we also elucidated the possible mechanism of miR-101-3p in children with IS. Therefore, we first certified that miR-101-3p may be a biological target for IS therapy.

## 2 Materials and methods

### 2.1 Animals

C57BL/6 young mice (male, clean class, 10-week-old) were purchased from Laboratory Animal Center, Guangzhou University of Chinese Medicine. All young mice were routinely fed and were housed at room temperature ranging from 22 to 24°C and lighting 12 h a day. After feeding for a week, the young mice were used for our experiments.

### 2.2 tMCAO model

tMCAO was established with C57BL/6 young mice by referring to the Longa method [28]. After fasting for 6–8 h, young mice were drugged using 1% pentobarbital sodium (0.45 mL/100 g, intraperitoneal injection). We fixed the limbs and head of young mice in the supine position and cut off the anterior neck hair. After disinfection, we made a midline incision of 1 cm, separated the muscle and fascia along the inner edge of the sternocleidomastoid muscle, and isolated the external carotid artery (ECA), internal carotid artery (ICA), and right common carotid artery (CCA). Then, we ligated the ECA distal end and CCA and coagulated the ECA distal end. Ophthalmic scissors were used to make a small cut at an angle of 45° in ECA. An ophthalmic tweezer was used to insert the thrombus slowly into the ICA from the incision until resistance appeared, indicating that the thrombus had reached the anterior cerebral artery (ACA). The proximal end of the ECA was ligated, the inserted thread was fixed, and the young mice were placed on a heating plate (temperature was controlled at 27–30°C) to ensure that the anal temperature reached 37°C. After 60 min of ischemia, the embolus was gently pulled out, the ECA was ligated, the thread at CCA was loosened to restore the blood flow for 12 h, and the incision was sutured. After disinfection, the young mice were routinely raised in cages. A similar procedure as the model group was performed with the sham group, except that the thrombus was not inserted.

### 2.3 Animal grouping

For HDAC9 overexpression, the head of young mice was attached to a stereotaxic instrument and the scalp was cut open to expose the skull after anesthesia. A small hole was made using a bone drill needle at the insertion points of the lateral ventricle (coordinates: from Bregma, 0.8 mm anterior; from midline, 0.5 mm left; from the surface: 2.5 mm ventra). Lentivirus-packed HDAC9 (2 μL, Hanbio, China) was injected into the lateral ventricle with a micro-injection pump for 10 min. Then, the needle was removed after 2 min and the scalp was sutured. Later, the young mice were routinely fed and subjected to a follow-up experiment after 3 weeks. For miR-101-3p overexpression, miR-101-3p agomiR (Genepharma, China) was dissolved in artificial cerebrospinal fluid (20 μmoL/L). Three days before modeling, young mice were injected into the lateral ventricle at a rate of 1 μL/min (5 μL in total). The model young mice were randomly divided into five groups: tMCAO group (*n* = 10), tMCAO + NC (*n* = 10), tMCAO + miR-101-3p (*n* = 20), tMCAO + miR-101-3p + vector (*n* = 10), and tMCAO + miR-101-3p + HDAC9 (*n* = 10).

### 2.4 2,3,5-Triphenyl tetrazolium chloride (TTC) staining

The young mice were decapitated, and the brain was frozen at −20°C for 10 min after removing the olfactory bulb, cerebellum, and lower brain stem. The brain was sectioned into six slices at 2 mm thickness in coronal. The brain slice was placed in 1% TTC (Sigma) at 37°C, and turned every 3 min to make it evenly exposed to the solution. The brain slices were removed and fixed with 10% paraformaldehyde for 2 h when the normal tissue was red and the infarcted tissue was white. Then, the brain slices were photographed and the infarct area was tested using Image-Pro Plus.

### 2.5 RNA extraction and real-time quantitative PCR (RT-qPCR)

To the brain tissue of each group (10 mg), 500 μL of Trizol (Invitrogen Life Technologies, USA) was added and homogenized in a homogenizer. Total RNA was determined by conventional methods and reversely transcribed using a Reverse Transcription kit (Takara, China) under the following conditions: 37°C for 15 min and 85°C for 5 s. Then, an amplification reaction was conducted using SYBR green PCR Master Mix (Applied Biosystems). The results were analyzed using the 2^−△△Ct^ method.

### 2.6 Western blot

The brain tissue was homogenized by adding RIPA buffer at a ratio of 1:10 (mass/volume). The protein concentration was determined using the bicinchoninic acid method, and the protein was pre-denatured by adding boiling water for 5 min. Protein samples (40 μg) were subjected to electrophoresis with 10% SDS-PAGE and transferred to a polyvinylidene difluoride membrane (Millipore, USA). After sealing with 5% skim milk for 1 h, the membranes were exposed to primary antibodies (anti-HDAC9 and anti-GAPDH) overnight at 4°C, followed by secondary antibodies for 1 h. The proteins were stained with enhanced chemiluminescence substrates for 1 min, and the western blot was exposed.

### 2.7 TUNEL staining

After dewaxing and hydration, the brain slices of each group were processed with 20 μg/mL protease K (Roche) to remove tissue proteins. Then, 100 μL of TUNEL mixture (Roche) was added to the slices at 37°C for 1 h. After the reaction was terminated, the slices were sealed with endogenous peroxidase and immersed in 0.3% H_2_O_2_ for 5 min. After washing, the slices were treated with a series of reagents, including horseradish peroxidase for 30 min, 3,3′-diaminobenzidine for 10 min, hematoxylin, gradient ethanol for dehydration, rendered transparent with xylene, and neutral gum for sealing. The TUNEL-positive cells were observed and photographed with a fluorescence microscope (Nikon, Japan).

### 2.8 Nissl staining

After processing with xylene and gradient ethanol, the brain tissue slices were immersed in Nissl solution (Beyotime) for 10 min. After washing, the slices were dehydrated with gradient alcohol, rendered transparent with xylene, and sealed with neutral resin. Nissl bodies were photographed with a microscope. ImageJ software was applied to count the number of positive Nissl bodies per unit area.

### 2.9 Statistical analysis

All data were displayed as mean ± SD from three repetitions. Statistical analysis was performed using SPSS21.0 software (SPSS Inc, Chicago, IL, USA) with one-way ANOVA, and *P* < 0.05 was considered significant.


**Ethical approval:** The research related to animal use has complied with all the relevant national regulations and institutional policies for the care and use of animals.

## 3 Results

### 3.1 miR-101-3p was downregulated and HDAC9 was upregulated in tMCAO young mice

**Figure 1:**
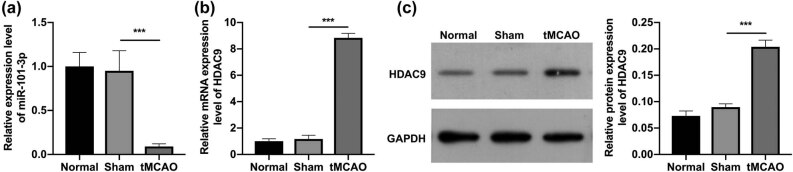
miR-101-3p was downregulated and HDAC9 was upregulated in tMCAO young mice. (a) RT-qPCR results of the change in miR-101-3p expression in the brain tissues of tMCAO young mice. (b) RT-qPCR analysis of HDAC9 expression in the brain tissues of tMCAO young mice. (c) The expression change of HDAC9 in tMCAO young mice was also tested with western blot. ***P < 0.001.

We first identified the changes in miR-101-3p and HDAC9 expression in brain tissues of tMCAO young mice. As displayed in Figure 1a, miR-101-3p expression in the tMCAO group was memorably lower than that in the sham group, indicating the downregulation of miR-101-3p in tMCAO young mice. And RT-qPCR data found that the expression of HDAC9 mRNA in the tMCAO group was signally higher than that in the sham group (Figure 1b). Simultaneously, we discovered that the level of HDAC9 protein was also observably increased in the tMCAO group versus that in the sham group (Figure 1c). In summary, these findings indicated that the construction of tMCAO young mice can cause downregulation of miR-101-3p and upregulation of HDAC9 in the brain tissues.

### 3.2 miR-101-3p overexpression reduced the infarct area in the brain of tMCAO young mice

**Figure 2:**
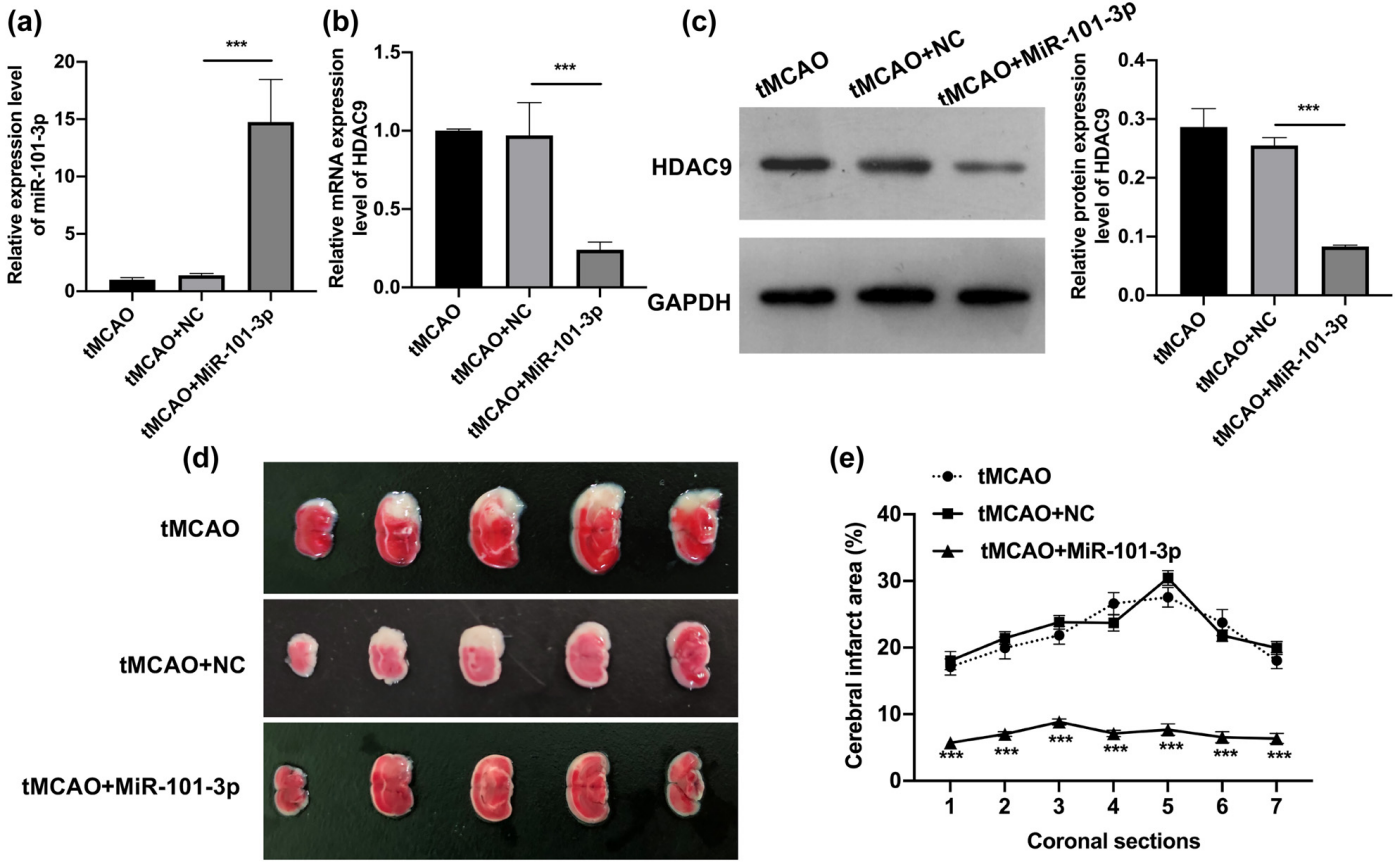
miR-101-3p overexpression reduced the infarct area in the brain of tMCAO young mice. (a) RT-qPCR was adopted to show the expression change of miR-101-3p in miR-101-3p-overexpressed tMCAO young mice brains. RT-qPCR (b) and Western blot (c) were applied to identify the influence of miR-101-3p overexpression on the level of HDAC9 in the brains of tMCAO young mice. (d) After miR-101-3p overexpression, the cerebral infarct area was confirmed with TTC staining in the coronal sections of tMCAO young mice brains. (e) The cerebral infarct area (%) was calculated and counted. ***P < 0.001.

Subsequently, we overexpressed miR-101-3p in tMCAO young mice by injecting miR-101-3p agomiR in the lateral ventricle. The RT-qPCR data indicated that after miR-101-3p overexpression, miR-101-3p was prominently upregulated in the brains of tMCAO young mice (Figure 2a). Meanwhile, the data manifested that miR-101-3p overexpression also could cause a remarkable downregulation of HDAC9 in the brains of tMCAO young mice (Figure 2b and c). Besides, our data indicated that the elevation of the cerebral infarction area in tMCAO young mice could be dramatically weakened by injecting miR-101-3p agomiR (Figure 2d and e). Thus, the data proved that we successfully overexpressed miR-101-3p in the lateral ventricle of tMCAO young mice and confirmed that the upregulation of miR-101-3p could reduce the cerebral infarction area in tMCAO young mice.

### 3.3 miR-101-3p overexpression improved neuronal morphology and attenuated neuronal apoptosis in tMCAO young mice

**Figure 3:**
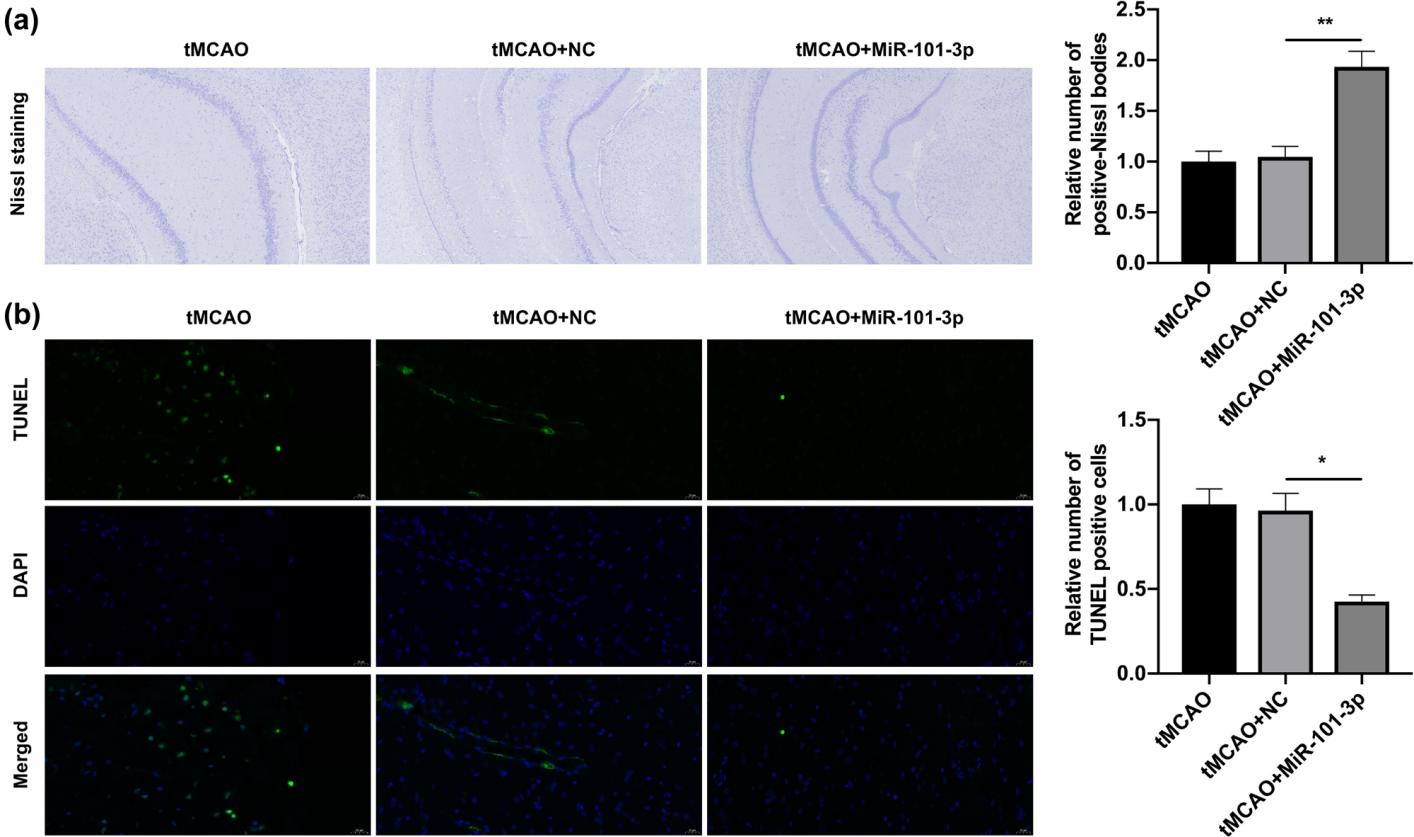
miR-101-3p overexpression improved neuronal morphology and attenuated neuronal apoptosis in tMCAO young mice. (a) Nissl staining was conducted to assess the impact of miR-101-3p overexpression on neuron morphology in the brain of tMCAO young mice. (b) After miR-101-3p overexpression, neuron apoptosis was shown through TUNEL staining in the brain of tMCAO young mice. Magnification, 200×, scale bar = 50 μm. *P < 0.05 and **P < 0.01.

We further investigated the influence of miR-101-3p overexpression on neuronal morphology and neuronal apoptosis in tMCAO young mice. The Nissl staining results indicated that in tMCAO and tMCAO + NC groups, the neurons showed obvious wrinkles, irregular shape, shallow staining, and a small number of Nisseni bodies; in the tMCAO + miR-101-3p group, the neuronal morphology was evidently improved, the neuronal morphology was fuller, and the number of Nisseni bodies was markedly higher than that of the tMCAO model group (Figure 3a). Moreover, the TUNEL staining results also exhibited that the number of TUNEL-positive cells was significantly reduced in the tMCAO + miR-101-3p group relative to that in tMCAO + NC groups (Figure 3b). Consequently, these data show that overexpression of miR-101-3p could downregulate HDAC9, ameliorate neuronal morphology and apoptosis in tMCAO young mice.

### 3.4 HDAC9 overexpression reversed the reduction of the cerebral infarction area mediated by miR-101-3p in tMCAO young mice

**Figure 4:**
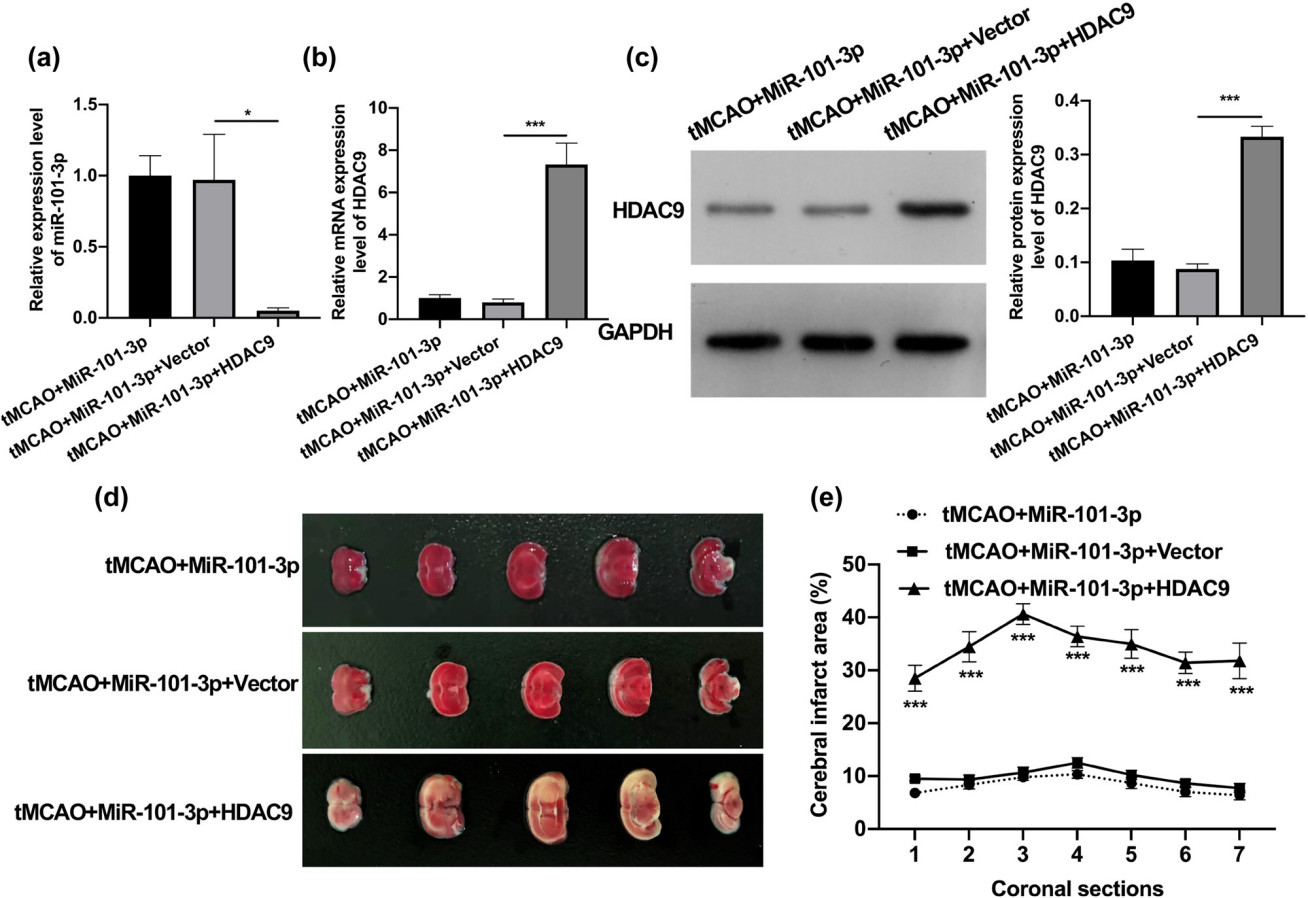
HDAC9 overexpression reversed the reduction of the cerebral infarction area mediated by miR-101-3p in tMCAO young mice. (a) RT-qPCR analysis of miR-101-3p in tMCAO young mice after miR-101-3p and/or HDAC9 overexpression. HDAC9 expression was tested by applying RT-qPCR (b) and Western blot (c) in tMCAO young mice after transfection with miR-101-3p agomiR and/or HDAC9 overexpression plasmid. (d) TTC staining was utilized to examine the effect of HDAC9 overexpression on the cerebral infarct area of miR-101-3p-overexpressed tMCAO young mice. (e) The cerebral infarct area was counted based on the TTC staining results. *P < 0.05 and ***P < 0.001.

Based on the above results, miR-101-3p could lower HDAC9 expression. We further investigated whether the neuroprotective effect of miR-101-3p on IS young mice was realized by downregulating HDAC9. We further overexpressed HDAC9 in miR-101-3p agomiR-injected tMCAO young mice. The RT-qPCR results first displayed that HDAC9 overexpression could visibly decrease miR-101-3p expression in miR-101-3p-overexpressed tMCAO young mice (Figure 4a). We also testified that the decrease in HDAC9 expression mediated by miR-101-3p agomiR could be notably reversed by HDAC9 overexpression in tMCAO young mice (Figure 4b and c). Meanwhile, TTC staining data indicated that HDAC9 overexpression could memorably attenuate the reduction of the cerebral infarction area, which was mediated by miR-101-3p agomiR in tMCAO young mice (Figure 4d and e). These results revealed that miR-101-3p overexpression could decrease the cerebral infarction area of tMCAO young mice by downregulating HDAC9.

### 3.5 HDAC9 overexpression weakened the neuroprotective role of miR-101-3p in tMCAO young mice

**Figure 5:**
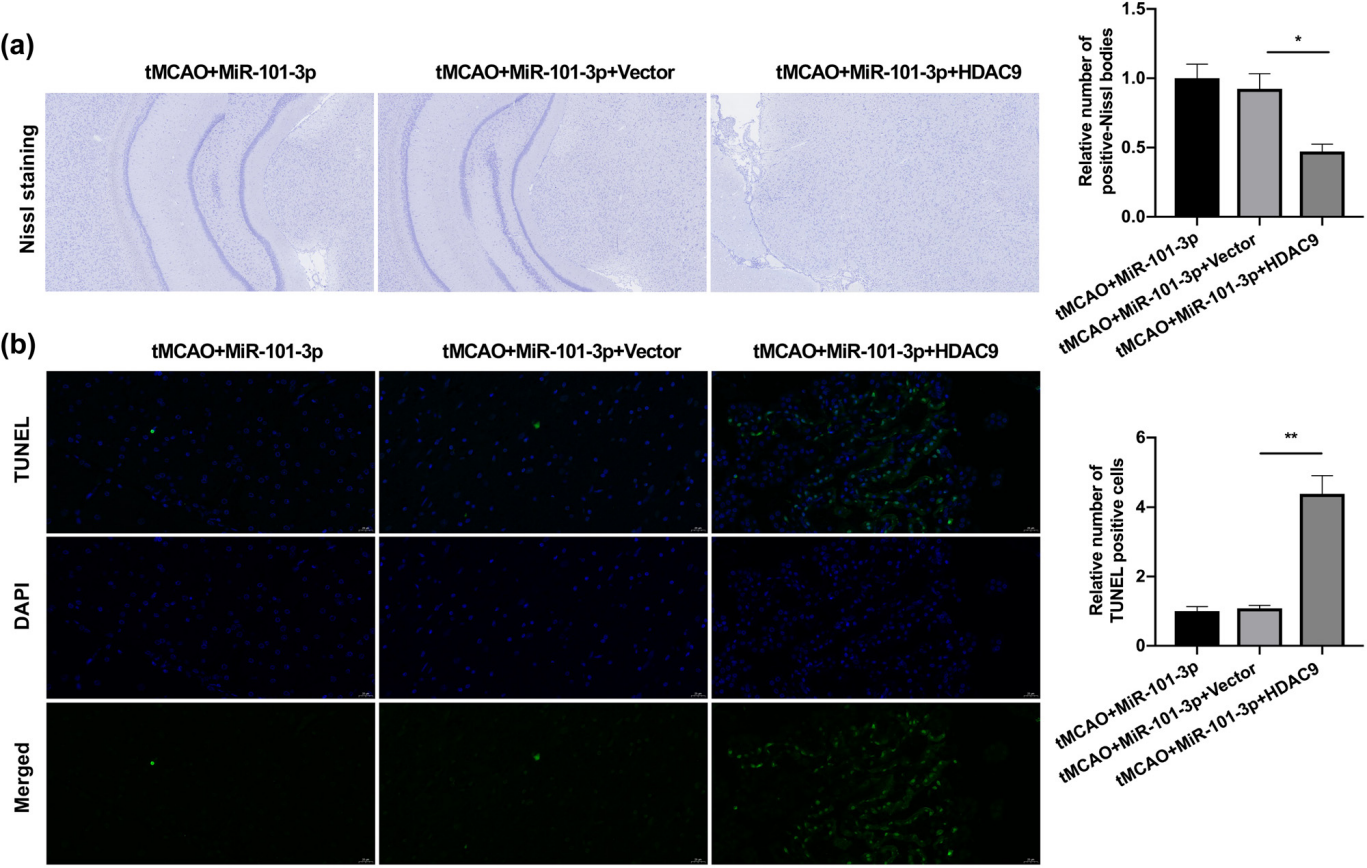
HDAC9 overexpression weakened the neuroprotective role of miR-101-3p in tMCAO young mice. (a) After miR-101-3p and/or HDAC9 overexpression, neuron morphology was monitored using Nissl staining in tMCAO young mice. (b) TUNEL staining was adopted to determine the influence of miR-101-3p and/or HDAC9 overexpression on the neuron apoptosis of tMCAO young mice. Magnification, 200×, scale bar = 50 μm. *P < 0.05 and **P < 0.01.

Finally, Nissl staining data further showed that compared with the miR-101-3p agomiR treatment group, the overexpression of HDAC9 could prominently reduce the number of Nisseni bodies and destroy the morphology of neurons (Figure 5a). Further, the TUNEL staining results also denoted that the decrease of TUNEL-positive cells, which was caused by miR-101-3p agomiR, also could be dramatically reversed by HDAC9 overexpression in tMCAO young mice (Figure 5b). Overall, we further verified that HDAC9 could participate in the protective process of miR-101-3p against IS in young mice.

## 4 Discussion

IS is a conventional neurological disease with a high disability rate around the world [29]. Currently, it is generally considered that reperfusion of blood flow in the ischemic site is an effective means to treat IS patients. Although timely thrombolytic therapy can recanalize blocked vessels, it has distinct limitations. On the one hand, reperfusion of blood flow can restore the function of the ischemic site and relieve the disease. On the other hand, it is easy to cause IRI and further aggravate the condition [30]. IS is also a key cause of disability in children, including epilepsy, paralysis, cognitive, and language impairment, which can cause permanent disability [31]. Therefore, an in-depth study on the pathogenesis of IRI in children is of great importance to find effective targets for IS treatment.

MiRNAs are known to play key roles in multiple physiological processes such as embryogenesis, immune system development, cell proliferation, apoptosis, etc. [9,32]. Moreover, multiple miRNAs have been certified to influence the occurrence and development of IS, including miR-126 [15], miR-191 [16], miR-130a [18], and miR-221 [33]. This study first established tMCAO in young mice in line with previous studies. Our results show that the infarct volume was increased most significantly in tMCAO young mice with 12 h reperfusion, which was chosen as the best tMCAO model. Next, we discovered that miR-101-3p was downregulated in tMCAO young mice. MiR-101-3p, discovered in recent years, mainly plays a crucial role in cancer progression and also affects neuroinflammation and neuronal damage [34,35,36]. Thus, we further speculated that miR-101-3p might alleviate IS in children. Our current results also proved that overexpression of miR-101-3p could alleviate neuron injury and suppress neuronal apoptosis in tMCAO young mice.

Moreover, we also testified that HDAC9 was highly expressed in tMCAO young mice. At present, increasing attention has been devoted to the function played by epigenetics in IRI [37,38]. Epigenetics mainly comprise DNA methylation, histone modification, and post-transcriptional regulation by non-coding RNAs including miRNA [39]. There are also multiple frequent histone modifications, such as acetylation, ubiquitin, carboxylation, methylation, glycosylation, phosphorylation, etc. [40]. Acetylation modification, as a key mechanism of gene regulation, is mainly regulated by histone acetyltransferases (HATs) and histone deacetylases (HDACs). The former can activate specific genes to induce transcription, while the latter can restrain transcription by preventing the binding of gene promoters to transcription regulatory elements [41]. Depending on different structures and expression forms, HDACs can be grouped into four families. Among them, the members of class II HDACs have tissue expression specificity and can also regulate the transcription and translation of target genes after experiencing various stimuli [42]. HDAC9, a member of class II HDACs, is mainly highly expressed in skeletal muscles and the brain [43]. HDAC9 has also been reported to affect the prognosis of various cancers by accelerating cell proliferation and blocking cell apoptosis [44,45]. The literature also shows that HDAC9 is relevant to cerebrovascular diseases such as atherosclerosis [46]. Encouragingly, studies have also shown that HDAC9 is associated with the risk of stroke [47–49], while the action and mechanism of HDAC9 are not fully understood. Our results also further showed that overexpression of HDAC9 could reverse the impacts of miR-101-3p on neuronal morphology and apoptosis in tMCAO young mice, indicating that miR-101-3p could relieve IS through HDAC9 in tMCAO young mice.

## 5 Conclusions

In the current study, we suggested that miR-101-3p was lowly expressed, while HDAC9 was highly expressed in tMCAO young mice, and miR-101-3p overexpression could downregulate HDAC9. Besides, we proved that miR-101-3p could ameliorate neuronal morphology and prevent neuronal apoptosis in tMCAO young mice by reducing HDAC9 expression. Therefore, our results manifested that miR-101-3p and HDAC9 might be the targets for IS therapy in children. Nevertheless, there are still certain limitations in our current results, for instance, whether miR-101-3p and HDAC9 can be directly combined and affect each other; the pathways through which HDAC9 can affect IS progression in children also need to be further investigated.
